# Atomically precise silver-based bimetallic clusters for electrocatalytic urea synthesis

**DOI:** 10.1093/nsr/nwae440

**Published:** 2024-11-29

**Authors:** Hong Chen, Lin Liu, Xiao-Hong Ma, Su-Jun Zheng, Xiao-Yu Dong, Ren-Wu Huang, Zhao-Yang Wang, Jinmeng Cai, Shuang-Quan Zang

**Affiliations:** Henan Key Laboratory of Crystalline Molecular Functional Materials, College of Chemistry, Zhengzhou University, Zhengzhou 450001, China; Henan Key Laboratory of Crystalline Molecular Functional Materials, College of Chemistry, Zhengzhou University, Zhengzhou 450001, China; Henan Key Laboratory of Crystalline Molecular Functional Materials, College of Chemistry, Zhengzhou University, Zhengzhou 450001, China; Henan Key Laboratory of Crystalline Molecular Functional Materials, College of Chemistry, Zhengzhou University, Zhengzhou 450001, China; Henan Key Laboratory of Crystalline Molecular Functional Materials, College of Chemistry, Zhengzhou University, Zhengzhou 450001, China; Henan Key Laboratory of Crystalline Molecular Functional Materials, College of Chemistry, Zhengzhou University, Zhengzhou 450001, China; Henan Key Laboratory of Crystalline Molecular Functional Materials, College of Chemistry, Zhengzhou University, Zhengzhou 450001, China; Henan Key Laboratory of Crystalline Molecular Functional Materials, College of Chemistry, Zhengzhou University, Zhengzhou 450001, China; Henan Key Laboratory of Crystalline Molecular Functional Materials, College of Chemistry, Zhengzhou University, Zhengzhou 450001, China

**Keywords:** atomically precise, bimetallic clusters, electrocatalysis, C–N coupling, urea synthesis

## Abstract

Electrocatalytic urea synthesis from CO_2_ and nitrate holds immense promise as a sustainable strategy, but its complicated synthesis steps and controversial C–N coupling mechanism restrict the design of efficient catalysts. Atomically precise metal cluster materials are ideal model catalysts for investigating the C–N coupling issues. Here we synthesize two atomically precise bimetallic clusters, Ag_14_Pd(PTFE)_6_(TPP)_8_ and Ag_13_Au_5_(PTFE)_10_(DPPP)_4_, both with icosahedral cores and similar ligands. We demonstrate that both clusters have good performance for electrocatalytic urea synthesis, with the production rates at the maximum Faradaic efficiency of 143.3 and 82.3 mg h^−1^ g_cat_^−1^, respectively. Bimetallic structures can induce charge polarization at the active sites of metal clusters, thereby influencing the selectivity. In mechanistic investigations, we propose that *NOH and *COOH are the rate-limiting steps for the reduction of nitrate and CO_2_, respectively, and that the key intermediates formed thereafter can significantly affect the C–N coupling process. This approach offers a deep understanding into C–N coupling through the utilization of atomically precise metal clusters.

## Teaser

The charge polarization of the active sites of the Ag-based clusters is induced by heterometallic doping, which regulates the reaction pathways and catalytic performance of the electrocatalytic urea synthesis.

## INTRODUCTION

Urea, a high-quality fertilizer with a nitrogen content of 46.7%, is also a crucial chemical raw material [1–3]. Currently, the predominant industrial route for urea synthesis is the Bosch–Meiser process, which relies on the thermochemical coupling of carbon dioxide (CO_2_) and ammonia (NH_3_) at high temperatures (150–200°C) and pressures (150–250 bar) [4–6]. This process consumes 80% of the globally produced NH_3_ from the Haber-Bosch process, necessitates significant fossil fuel consumption, and generates substantial greenhouse gas emissions [7–11]. Consequently, exploring and developing a green and sustainable method for urea synthesis at ambient temperature and pressure is paramount [12]. Electrocatalytic co-reduction of CO_2_ and nitrate (NO_3_^−^) under environmental conditions offers a promising solution for carbon/nitrogen neutralization and environmental pollution alleviation [12–15]. However, the exploration of urea production through the above process has been hampered by the complex 16-electron reduction process (2NO_3_^−^ + CO_2_ + 18H^+^ + 16e^−^ → NH_2_CONH_2_ + 7H_2_O) [16,17]. In particular, a comprehensive understanding of the mechanism of C–N bond formation in electrocatalysis and the structural design of catalysts remain fundamental challenges.

Ligand-protected coinage-metal clusters contain a series of fascinating structures, which have attracted great attention in the field of luminescence and catalysis due to their precise and tunable structures [18–20]. Besides, the high atomic utilization rate, the high proportion of low-coordinated atoms, and distinctive surface structures enable atomic-level investigations of the cluster-based catalytic processes, thereby providing an excellent platform to delve into the structure–catalytic performance relationship [21–25]. Currently, silver clusters have shown impressive catalytic performance and selectivity in reactions such as the electrocatalytic NO_3_^−^ reduction reaction (NO_3_RR) and the electrocatalytic CO_2_ reduction reaction (CO_2_RR) [26–28]. Using atomically precise silver clusters to couple the two aforementioned reaction processes and promote the C–N coupling for urea synthesis holds significant importance in understanding the reaction mechanisms and guiding the design of more efficient catalysts. Additionally, since the electrocatalytic synthesis of urea involves the co-adsorption of C-containing and N-containing intermediates to promote the C–N coupling process, the design of dual active sites of catalysts is crucial for enhancing the performance and selectivity of urea synthesis [14,29].

Herein, we have designed and synthesized two atomically precise silver-based bimetallic clusters, namely Ag_14_Pd(PTFE)_6_(TPP)_8_ (Ag_14_Pd) and Ag_13_Au_5_(PTFE)_10_(DPPP)_4_ (Ag_13_Au_5_). By introducing Pd and Au atoms, we can effectively regulate the charge polarization of the active sites on the cluster catalysts, leading to changes in product distribution and ultimately improving the Faradaic efficiency (FE) and yield of urea. Both clusters show good performance for electrocatalytic urea synthesis, with the production rates at the maximum FE being 143.3 and 82.3 mg h^−1^ g_cat_^−1^, respectively. Therefore, by using the atomically precise metal clusters as model catalysts, this work not only deepens our fundamental understanding of the C–N bond formation, but also opens new possibilities for the development of advanced cluster-based catalysts for urea synthesis.

## RESULTS AND DISCUSSION

### Synthesis and crystal structures

Both Ag_14_Pd and Ag_13_Au_5_ nanoclusters were synthesized via a one-pot method. The detailed synthetic procedures are given in the Supplementary data. Briefly, The Ag_14_Pd cluster was synthesized by reducing a mixture of AgNO_3_, Pd(PPh_3_)_4_, triphenylphosphine (TPP) and pentafluorothiophenol (PTFE) with NaBH_4_ in a mixture of three different solvents of methanol (MeOH), dichloromethane (DCM) and water. Red single crystals were grown by diffusion with hexane after one day (Fig. S1a). By contrast, the synthesis of Ag_13_Au_5_ clusters was carried out by directly reducing silver and gold precursors with NaBH_4_ in CH_2_Cl_2_ and acetone. In a typical synthesis procedure, Ag_13_Au_5_ was prepared via the reduction of a mixture of CF_3_COOAg, 1,3-bis(diphenylphosphino)propane (DPPP), PTFE and chloro(dimethyl sulfide)gold(I) (Me_2_SAuCl) at room temperature. After one week, the red crystals of Ag_13_Au_5_ were afforded through solvent diffusion methods (Fig. S1b).

**Figure 1.**
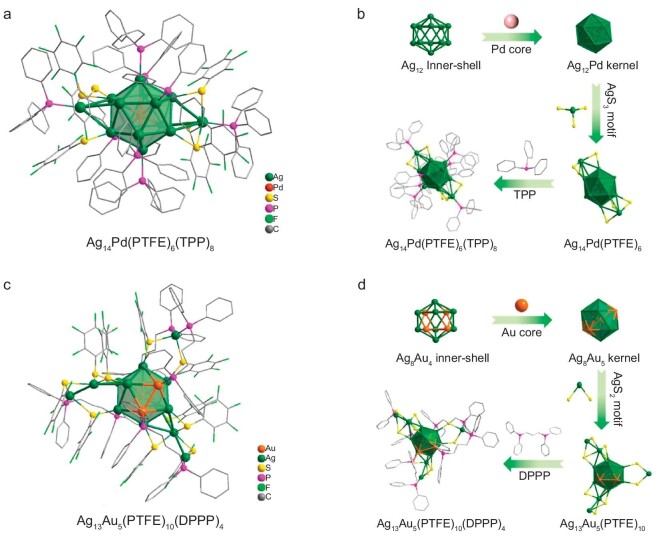
Crystal structures. (a) Total molecular structure of the Ag_14_Pd cluster. (b) The structural evolution of Ag_14_Pd. (c) Total molecular structure of the Ag_13_Au_5_ cluster. (d) The structural evolution of Ag_13_Au_5_.

The single crystal structural analysis reveals that the Ag_14_Pd crystallizes in the monoclinic space group (*I*2*/a*). As shown in Fig. 1a and b, the overall structure of Ag_14_Pd consists of an inner Pd@Ag_12_ centered icosahedron oppositely capped by two AgS_3_P units and co-stabilized by eight TPP. The Ag–Ag separations in the Ag_12_ icosahedron range from 2.85 Å to 2.96 Å, while the Ag–Ag distances between the AgS_3_P motif and Ag_12_ icosahedron fall in the range of 3.22–3.32 Å, both indicating the existence of argentophilic interactions. The two outer Ag atoms were each connected to the Ag_12_ icosahedron through Ag–Ag bonds with an average distance of 3.28 Å, further consolidated by three bridging thiolate ligands. The Ag–S bond lengths are in the range of 2.44–2.68 Å. Two of the eight TPP anchor to the outer Ag atoms, while the remaining six are bonded to the Ag atoms on the equatorial plane.

Ag_13_Au_5_ crystallizes in the centrosymmetric monoclinic space group (*P*2_1_/*c*). As shown in Fig. 1c and d, the overall structure of Ag_13_Au_5_ consists of an inner Au@Au_4_Ag_8_ centered icosahedron capped by two Ag_2_S_4_P units and one AgS_2_P_2_ unit, and the central Au atom is coplanar with the four Au atoms on the icosahedron fixed by four DPPP. In the Au_4_Ag_8_ icosahedron, the Au–Au bond lengths are in the range of 2.705–2.862 Å, the Au–Ag bond lengths are in the range of 2.768–2.989 Å, and the Ag–Ag bond lengths are in the range of 2.851–2.995 Å. The Ag_2_S_4_P unit is connected with the Au_4_Ag_8_ icosahedron via Ag–Ag, while the AgS_2_P_2_ unit is connected with the Au_4_Ag_8_ icosahedron via Ag–S–Ag, and is co-stabilised by bidentate phosphine ligands linked to an Au atom in the icosahedron and to an Ag atom in the Ag_2_S_4_P or AgS_2_P_2_ units. All silver atoms are consolidated by bridging thiolate ligands, the Ag–S bond lengths are in the range of 2.461–2.937 Å. Although the individual Ag_13_Au_5_ cluster is chiral, it contains two pairs of enantiomers in each cell, so the cluster as a whole is racemic (Fig. S2).

### Microstructure and morphology analyses

**Figure 2.**
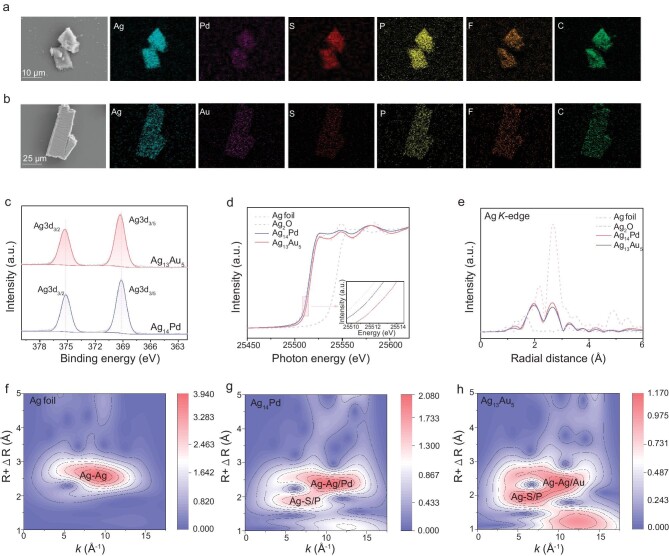
Structural characterization. EDS elemental mapping images of (a) Ag_14_Pd, and (b) Ag_13_Au_5_ clusters. (c) Ag 3d XPS profiles of the clusters. (d) Normalized XANES spectra of the Ag *K*-edge for Ag_14_Pd and Ag_13_Au_5_ clusters. (e) *k*^3^-weighted χ(*k*) function of the EXAFS spectra of the Ag *K*-edge for Ag_14_Pd and Ag_13_Au_5_ clusters. Ag foil and Ag_2_O were used as standard samples. WT contour plots of the *k*^3^-weighted EXAFS data of (f) Ag foil, (g) the Ag *K*-edge of Ag_14_Pd cluster, and (h) the Ag *K*-edge of Ag_13_Au_5_ cluster.

Scanning electron microscopy (SEM) and related energy dispersive X-ray spectroscopy (EDS) further demonstrated the element compositions of Ag_14_Pd and Ag_13_Au_5_ (Fig. 2a, b). The Ag_14_Pd cluster contains Ag, Pd, S, P, F, and C elements, and the Ag_13_Au_5_ cluster contains Ag, Au, S, P, F, and C elements. Both of them exhibit a uniform distribution of elements and no other impurities are present. The organic solutions of both clusters were detected by ultraviolet-visible spectroscopy (UV-vis, Fig. S3). At around 250 nm, both clusters have a broad peak with a similar shape, representing the absorption vibrational peak of the ligands. Besides, the Ag_14_Pd cluster has a shoulder peak around 310 nm and two major absorption peaks at 422 and 508 nm. The Ag_13_Au_5_ cluster has three major absorption peaks at 369, 387 and 485 nm, and two shoulder peaks at 340 and 445 nm. This indicates that atom doping significantly affects the electronic structure.

X-ray photoelectron spectroscopy (XPS) demonstrated that the doping of the heterometallic atoms caused a difference in the electronic structure of the two clusters [30–32]. As shown in Fig. 2c, Ag_14_Pd exhibits Ag 3d_3/2_ and Ag 3d_5/2_ peaks at 375.2 and 369.1 eV, respectively. In contrast, the Ag peaks for the Ag_13_Au_5_ cluster appear at higher binding energies, with Ag 3d_3/2_ and Ag 3d_5/2_ peaks at 375.3 and 369.3 eV, respectively. This observation indicates the varying degrees of electronic perturbation experienced by the silver clusters as a result of doping with heterometallic atoms [33,34]. Concretely, the Ag in the bimetallic clusters becomes more positively charged via electron donation from Ag to the dopant, resulting in a decrease in electron density of the Ag atoms [35]. Moreover, the XPS data of the dopants were also studied (Fig. S4). Regardless of the dopants being Au or Pd, a similar trend was found, that is, Au 4f, and Pd 3d binding energies were positively shifted for those of bulk metals [36,37]. These findings further confirm the existence of strong interactions between the dopant and the external Ag atoms, leading to different electronic states.

To further analyze the coordination environment, Ag_14_Pd and Ag_13_Au_5_ clusters were characterized by X-ray absorption fine structure (XAFS) spectroscopy. Fig. 2d shows the Ag *K*-edge X-ray absorption near-edge structure (XANES) spectra of Ag_14_Pd and Ag_13_Au_5_ in reference to Ag_2_O and Ag foil. In the Ag *K*-edge XANES spectra, the white-line peak position of the Ag *K*-edge of Ag_14_Pd and Ag_13_Au_5_ within the range of 25 500 to 25 515 eV is slightly higher than that of Ag foil [38]. This further confirms that the average valence state of the Ag atoms in the two clusters is between 0 and +1. In addition, the near-edge absorption peak of Ag_14_Pd was found to be located lower than Ag_13_Au_5_, suggesting that the average valence state of Ag in Ag_14_Pd is lower than that of Ag atoms in Ag_13_Au_5_ [39]. To quantitatively evaluate the valence states of Ag in Ag_14_Pd and Ag_13_Au_5_, the nearly linear portion of the rising *K*-edge was selected for integration and the integrated average intensity was defined as the *K*-edge position [40,41]. Subsequently, the valence states of Ag in Ag_14_Pd and Ag_13_Au_5_ were quantitatively calculated according to the *K*-edge position, which was Ag^0.04+^ and Ag^0.09+^ for Ag_14_Pd and Ag_13_Au_5_, respectively (Fig. S5). The Pd *K*-edge XANES spectra of Ag_14_Pd and Au *L*_3_-edge XANES spectra of Ag_13_Au_5_ are shown in Fig. S6a and b. The radial structure functions (RSFs) of the extended X-ray absorption fine structure (EXAFS) spectra of the clusters are illustrated in Fig. 2e and Fig. S6c, d. It is worth noting that the interatomic distance of the Ag–Ag bond in Ag_14_Pd (2.64 Å) and Ag_13_Au_5_ (2.66 Å) is slightly shorter than that of Ag foil (2.68 Å) due to the formation of Ag–Pd/Au bonding. Then, wavelet transform (WT) analysis of the Ag *K*-edge EXAFS oscillations of Ag_14_Pd and Ag_13_Au_5_ was performed (Fig. 2f, g and Fig. S7). The WT contour plots of Ag_14_Pd and Ag_13_Au_5_ resolve the Ag–Ag/Pd and Ag–Ag/Au bonds, which also confirmed the different coordination of Ag atoms between the Ag_14_Pd and Ag_13_Au_5_ samples [42–44].

### Electrochemical urea synthesis performance

**Figure 3.**
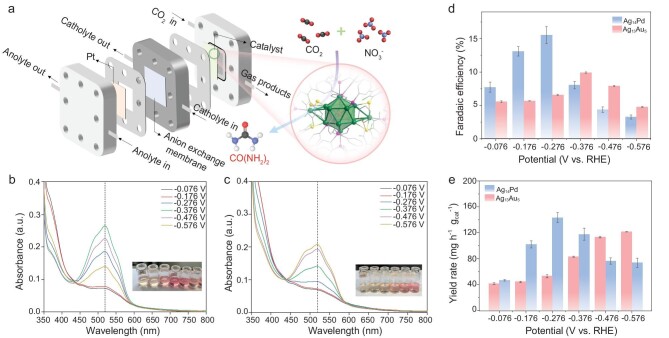
Electrochemical performance. (a) Schematic diagram of the flow cell. UV-vis spectra of post-reaction electrolyte after the diacetyl monoxime method at different potentials by using the (b) Ag_14_Pd cluster and (c) Ag_13_Au_5_ cluster as catalysts. Urea FEs and the urea yield rates of (d) Ag_14_Pd and (e) Ag_13_Au_5_ at different potentials.

A gas diffusion electrode (GDE)-based flow cell was used to evaluate the performance of Ag_14_Pd and Ag_13_Au_5_ for electrocatalytic urea synthesis (Fig. 3a). For the anode chamber, 50 mL of 1 M KOH solution was used as the electrolyte, while for the cathode chamber, 50 mL of 1 M KOH solution containing 200 ppm NO_3_^−^-N was used as the electrolyte. The prepared catalysts loaded on carbon paper were used as the GDE cathode to convert NO_3_^−^ and CO_2_ to urea. The anode is a Pt foil capable of promoting the oxygen evolution reaction. A linear sweep voltammetry (LSV) test was initially carried out to evaluate the current response for Ag_14_Pd and Ag_13_Au_5_ clusters. As shown in Fig. S8, when switching the atmosphere of the electrolyzer from Ar to CO_2_, the current densities of both clusters show significant differences. The results indicate that reactions related to CO_2_ reduction have occurred. Chronoamperometric tests (Fig. S9) were carried out in different potentials and high-purity CO_2_ was continuously bubbled into the cathodic cell during the experiments.

The concentration of urea was measured by the diacetyl monoxime method [1,7,45]. Besides urea, a series of byproducts, including ammonia (NH_3_), NO_2_^−^, CO and hydrogen (H_2_), were also identified through ion chromatography (IC) and gas chromatography (GC) analysis (Figs S10–S12). The absorbance of the solution at 525 nm was measured by a UV-vis spectrophotometer for the quantitative analysis of urea (Fig. 3b, c). Fig. 3d and e show the FEs and yield of urea on Ag_14_Pd and Ag_13_Au_5_ catalysts in the applied potential ranges of −0.076 to 0.576 V vs. reversible hydrogen electrode (RHE), respectively. The Ag_14_Pd catalyst exhibits its optimal performance in urea synthesis at a potential of −0.276 V vs. RHE, attaining a urea FE of 15.8% and a yield rate of ∼143.3 mg h^−1^ g_cat_^−1^. However, the Ag_13_Au_5_ catalyst achieves the best urea synthesis performance at a potential of −0.376 V vs. RHE, with a urea FE and yield rate of 9.7% and ∼82.3 mg h^−1^ g_cat_^−1^, respectively. The results showed that metal doping with Au or Pd had a significant effect on the performance. In addition, the catalytic product compositions and corresponding FEs of Ag_14_Pd and Ag_13_Au_5_ are illustrated in Fig. S13. Notably, the FEs of NO_2_^−^ are higher in the Ag_14_Pd cluster, while the FEs of CO are higher in the Ag_13_Au_5_ cluster. The above results indicate that the two clusters exhibit different catalytic selectivity towards the electrocatalytic synthesis of urea using NO_3_^−^ and CO_2_ as substrates. The Ag_14_Pd cluster prefers the NO_3_RR, while the Ag_13_Au_5_ cluster prefers the CO_2_RR. In 7 consecutive cycles at −0.276 V vs. RHE, the current density and urea yield rate remained almost unchanged (Figs S14–S16), indicating the excellent electrocatalytic stability of Ag_14_Pd and Ag_13_Au_5_. In addition, the UV-vis test results of the catalysts before and after the reaction were consistent, indicating that structural stability can be maintained during the process of electrocatalysis (Fig. S17). To further demonstrate the stability of the catalyst, the XPS of Ag_14_Pd and Ag_13_Au_5_ was measured after the catalytic reaction. The results showed that the metal valence of the Ag_14_Pd and Ag_13_Au_5_ catalysts remained unchanged after the reaction (Fig. S18). Since both Ag_14_Pd and Ag_13_Au_5_ exhibit low onset potentials in the electrocatalytic synthesis of urea, and their product selectivity differs significantly, it is compelling to gain a profound understanding of the C–N bond formation mechanism in both Ag_14_Pd and Ag_13_Au_5_ clusters. Additionally, elucidating the mechanism of NO_3_^−^ and CO_2_ co-reduction and the structure–activity relationship would be valuable, potentially contributing to the development of more advanced catalysts.

### *In situ* characterization and theoretical calculation

**Figure 4.**
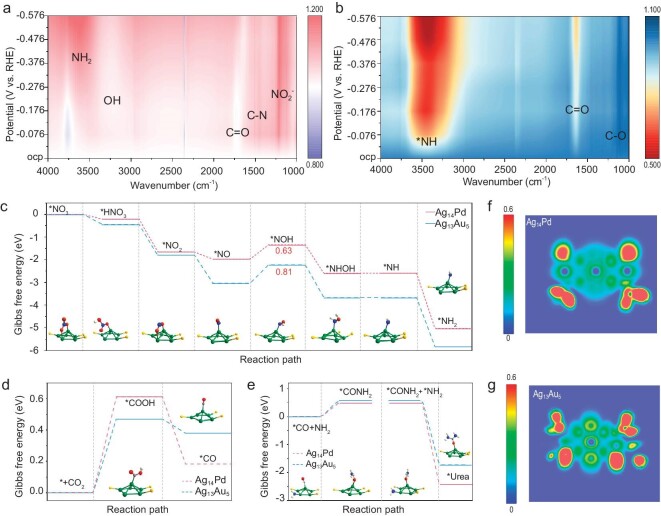
*In situ* characterization and DFT calculation. Potential-dependent *in situ* ATR-FTIR spectra of (a) Ag_14_Pd and (b) Ag_13_Au_5_. Free energy diagram for urea production on Ag_14_Pd and Ag_13_Au_5_: (c) NO_3_^−^ reduction, (d) CO_2_ reduction, (e) formation of urea from *NH_2_ and *CO. Two-dimensional electron local density maps of (f) Ag_14_Pd cluster and (g) Ag_13_Au_5_ cluster.

*In situ* Fourier-transform infrared (FTIR) spectroscopy analysis was used to identify intermediates produced on Ag_14_Pd and Ag_13_Au_5_ clusters during urea synthesis. As displayed in Fig. 4a, b and Fig. S19, when the applied potential increases from OCP to −0.576 V vs. RHE, two prominent characteristic peaks centered at 3758 cm^−1^ and 1504 cm^−1^ for Ag_14_Pd are observed. These peaks can be assigned to the accumulation of *NH_2_ intermediate species and the C–N bond vibration, respectively [46,47]. As the applied potential gradually increased, the C–N vibrational signals exhibited a corresponding increase, while the *NH_2_ peaks initially increased and then decreased. This trend suggests that *NH_2_ intermediates were initially generated and subsequently consumed during the reaction, concomitant with the occurrence of the C–N coupling process [48]. In addition, the peaks centered at 1208 cm^−1^ and 1650 cm^−1^ are attributed to NO_2_^−^ and C=O vibrational peaks, respectively [49,50]. The peak at 3437 cm^−1^ for the Ag_13_Au_5_ cluster can be assigned to the *NH intermediates [51]. Notably, the intensity of this peak gradually increased with the increase of applied potential, indicating that the *NH intermediates were accumulating on the catalyst surface. In addition, the peaks at 1625 cm^−1^ and 1112 cm^−1^ are attributed to the vibration of the C=O and C–O bond, respectively [52]. Compared to Ag_14_Pd, Ag_13_Au_5_ produced weaker signals for NO_2_^−^ peaks, but significantly stronger C=O signal peaks. This result further confirms that Ag_14_Pd is more prone to undergo the NO_3_RR, while Ag_13_Au_5_ clusters are more likely to engage in the CO_2_RR, which aligns with the performance test results. In the process of urea synthesis, the C–N coupling of *NH_2_ intermediates with *CO intermediates is crucial. In the Ag_14_Pd cluster, the *NH_2_ signal peaks first increase and then decrease, indicating that with the increase of potential, the *NH_2_ produced by the reaction initially accumulates on the surface and is then consumed during the C–N coupling reaction. In contrast, the *NH in the Ag_13_Au_5_ cluster continues to accumulate on the surface, suggesting that it is less consumed in the subsequent hydrogenation and C–N coupling processes. This also verifies that Ag_14_Pd clusters outperform Ag_13_Au_5_ clusters in the reaction for the synthesis of urea.

To further elucidate the underlying reasons for the effect of heterometallic doping modulating the properties of the active sites on the selectivity of the urea synthesis process, density functional theory (DFT) calculations were performed on the optimized Ag_14_Pd and Ag_13_Au_5_ clusters. Fig. 4c and e show the detailed free energy diagrams of the lowest energy pathways of Ag_14_Pd and Ag_13_Au_5_ clusters for NO_3_^−^ to *NH_2_, CO_2_ to *CO, *CO and *NH_2_ to urea. The reaction process from NO_3_^−^ to *NH_2_ is essentially thermodynamically spontaneous except for the *NO → *NOH step (Fig. 4c). The hydrogenation of *NO to form *NOH is considered to be the rate-limiting step in the reaction, with reaction energy barriers to overcome 0.63 and 0.81 eV for Ag_14_Pd and Ag_13_Au_5_ clusters, respectively. By contrast, CO_2_ was first physically adsorbed on the surface of Ag_14_Pd and Ag_13_Au_5_. The hydrogenation of *COOH is a potential rate-limiting step with reaction-free energy of 0.61 and 0.47 eV for Ag_14_Pd and Ag_13_Au_5_ clusters, respectively. Subsequently, *COOH undergoes spontaneous thermodynamic reduction to *CO. This indicates that in the co-reduction reaction of NO_3_^−^ and CO_2_, *NH_2_ is more easily generated on Ag_14_Pd, while CO* is more easily obtained on Ag_13_Au_5_, confirming the influence of charge-polarization on the selectivity of the catalytic process. Moreover, the C–N coupling process is crucial for urea synthesis. Energy barriers to be overcome by Ag_14_Pd and Ag_13_Au_5_ during the coupling reaction of *NH_2_ with *CO are 0.48 eV and 0.58 eV, respectively (Fig. 4e). This indicates that Ag_14_Pd is more conducive to C–N coupling reaction to form urea, which is consistent with experimental findings. This was also confirmed by the calculated adsorption energies of *NH_2_ and *CO intermediates for Ag_14_Pd and Ag_13_Au_5_ (*NH_2_ on Ag_14_Pd and Ag_13_Au_5_: −2.49 eV and −2.42 eV, *CO on Ag_14_Pd and Ag_13_Au_5_: −0.19 eV and 0.01 eV). Ag_14_Pd exhibits a higher adsorption capacity for the key intermediates involved in C–N coupling. Local charge density plots of Ag_14_Pd and Ag_13_Au_5_ (Fig. 4f, g) reveal that the electrons of the Ag atoms are delocalized and donated to the surrounding Pd or Au atoms, owing to the higher electronegativity of Pd and Au atoms. Bader charge analysis confirms that in the Ag_14_Pd cluster, the Ag atoms denote 0.08e^−^ to core Pd atoms, while in the Ag_13_Au_5_ cluster, the Ag atoms contribute 0.81e^−^ to core Au atoms. Despite these differences in electron donation, both Ag_14_Pd and Ag_13_Au_5_ maintain a balanced overall electron distribution (Fig. S20).

In brief, based on the *in situ* test characterizations and DFT calculation results, combined with the confirmation of reaction intermediates by *in situ* differential electrochemical mass spectrometry (DEMS, Fig. S21), the mechanism of Ag_14_Pd and Ag_13_Au_5_ for electrocatalytic synthesis of urea can be inferred as shown in Fig. S22. The electroreduction of NO_3_^−^ follows the N-alternating pathway: NO_3_^−^ → *HNO_3_ → *NO_2_ → *NO → *NOH → *NHOH → *NH → *NH_2_. The intermediate *COOH plays a pivotal role in the reduction of CO_2_ to *CO. Notably, the formation of *CONH_2_ through the C–N coupling between the *NH_2_ and *CO intermediates is intimately linked to urea selectivity.

## CONCLUSION

In summary, utilizing atomically precise metal clusters as catalysts has demonstrated significant advantages in studying the reaction process and mechanism of electrocatalytic urea synthesis. We have discovered that incorporating Pd or Au into the Ag cluster can markedly influence the catalytic reaction pathway, thereby altering the FEs of electrochemical co-reduction of CO_2_ and NO_3_^−^ to form urea. In a GDE-based flow cell under ambient conditions, the Ag_14_Pd cluster is more favorable for the NO_3_RR, while the Ag_13_Au_5_ cluster is more inclined towards CO_2_RR. The urea formation rate on Ag_14_Pd and Ag_13_Au_5_ clusters at their respective highest FEs are 143.3 and 82.3 mg h^−1^ g_cat_^−1^, respectively. Experimental and DFT results confirmed that *NH_2_ and *CO were the most likely C–N coupling intermediates, and verified the influence of charge polarization induced by hetero-metal doping on the selectivity of electrocatalytic urea synthesis. The findings not only provide strategies for access to desired bimetallic catalysts, but also establish a platform for the elucidation of the structure–activity relationship of silver-based bimetallic catalysts.

## Supplementary Material

nwae440_Supplemental_Files
