# Supplementary material for: Atomically precise silver-based bimetallic clusters for electrocatalytic urea synthesis
Source: Natl Sci Rev. 2024 Nov 29;12(2):nwae440. doi: 10.1093/nsr/nwae440 (PMC11740507; doi:10.1093/nsr/nwae440)
Supplement: nwae440_Supplemental_Files [file nwae440_supplemental_files.zip › 1228-Supporting_Materials.pdf]

Atomically Precise Silver-Based Bimetallic Clusters for Electrocatalytic Urea Synthesis

Hong Chen,[1,†] Lin Liu,[1,†] Xiao-Hong Ma,[1,†] Su-Jun Zheng,[1] Xiao-Yu Dong,[1] Ren-Wu Huang,[1] Zhao-Yang Wang,[1] Jinmeng Cai,[1,*] Shuang-Quan Zang[1,*]

[1]Henan Key Laboratory of Crystalline Molecular Functional Materials, College of Chemistry, Zhengzhou University, Zhengzhou 450001, China.

*Corresponding Authors: zangsqzg@zzu.edu.cn; caijinmeng@zzu.edu.cn

[†]These authors contributed equally to this work.

Contents

1. Experimental..S2

1.1 Reagents..S2

1.2 Apparatus..S2

1.3 Calculation procedure..S3

1.4 Synthesis..S3

1.5 Electrochemical measurements..S4

1.6 Products identification and quantification..S3

1.7 References..S6

2. Selected spectra and data referred in the paper..S7

1. Experimental

1.1 Reagents

All chemicals and solvents obtained from suppliers were used without additional purification. CF_3COOAg was prepared by reacting Ag_2O and CF_3COOH in pure water, while Me_2SAuCl was prepared by reacting dimethyl sulfide and $HAuCl_4 \cdot 4H_2O$ in EtOH.

1.2 Apparatus

Single-crystal X-ray diffraction (SCXRD) was performed on a Rigaku XtaLAB Pro diffractometer using Cu Kα radiation (λ = 1.54184 Å). Powder X-ray diffraction (PXRD) were collected on a Riguku D / Max-2500PC X-ray diffractometer. Morphology of all samples were carried out using Zeiss Sigma 500 on a scanning electron microscopy (SEM) measurement. X-ray absorption fine structure (XAFS) measurements were recorded at Shanghai Synchrotron Radiation Facility (SSRF). X-ray photoelectron spectroscopy (XPS) measurements were performed on a 5000 VersaProbe (PHI) instrument using Al Kα radiation as the excitation source. All spectra were calibrated using the C 1s signal at 284.6 eV due to adventitious hydrocarbons.

In situ FTIR measurements

The in situ FTIR tests were performed on a Bruker INVENIO S instrument. A three-electrode system was selected for testing in the external reflection mode. The reference electrode was an AgCl electrode, the counter electrode was a platinum wire, and the catalyst was drop-coated on monocrystalline silicon coated with a gold film as the working electrode. The reaction was carried out in a 1 M KOH solution containing 200 ppm NO_3^--N. During spectral detection, CO_2 was continuously passed as the gas phase reactant and the i-t curve was detected in constant potential intervals of 0.100, -0.076, -0.176, -0.276, -0.376, -0.476, -0.576 V vs. RHE, and the spectral acquisition was started after a stabilization time of 100 s at each potential.

In situ DEMS measurement

The in situ DEMS experiments were performed on a Linglu instrument equipped with a differential electrochemical mass spectrometer, which consisted of a high and a mild vacuum environment in the first and second chambers, respectively. A typical three-electrode cell was used for the measurements, with the cathode chamber directly connected to the mass spectrometer. $Ag_{14}Pd$ and $Ag_{13}Au_5$ loaded on carbon paper were prepared and then cut into circles with a diameter of 8 mm (similar to the size of the reaction cell) and fixed on the surface of the polytetrafluoroethylene membrane, which served as the working electrode for the tests. Finally, a CO_2-saturated 1 M KOH + 200 ppm NO_3^--N solution was used as the electrolyte. The CO, NO, and NH_3 generated during multiple LSV at 0.8~-1 V (vs. RHE) entered the first vacuum chamber of the mass spectrometer for further analysis.

1.3 Calculation procedure

The DFT calculations were performed using the Vienna ab initio Simulation Package (VASP) with the projector augmented-wave potentials.[1,2] The generalized gradient approximation (GGA) with the Perdew-Burke-Ernzerhof (PBE) functional was employed to describe the exchange-correlation.[3,4] A cutoff energy for the plane-wave basis was set to 420 eV for all calculations. The energy convergence criteria for self-consistent-field iteration was 10^{-5} eV, and the atomic positions were fully optimized until all the residual forces are smaller than 0.04 eV $Å^{-1}$. Firstly, the $Ag_{14}Pd$ and $Ag_{13}Au_5$ clusters were placed into large enough box with length more than 20 Å, respectively, and the structure optimation were performed. Then, the free energy differences of the CO_2 decomposition, NO_3 decomposition, and urea formation were calculated, repectively, on both of the $Ag_{14}Pd$ and $Ag_{13}Au_5$ clusters. For all the simulations, the DFT−D3 Grimme strategy for dispersion correction of total energy was used to include the vdW interactions.[5]

1.4 Synthesis

Synthesis of $Ag_{14}Pd(PTFE)_6(TPP)_8$

$AgNO_3$ (8.5 mg, 0.05 mmol) was dissolved in 2 mL of CH_3OH, and $Pd(PPh_3)_4$ (5 mg in 4 mL $CHCl_3$) was added dropwise to the above CH_3OH mixture solution with continuous stirring. The color of the solution changed from colorless to a clear brown within 5 min. Then 10 μL pentafluorothiophenol (PTFE) was added and the solution gradually became cloudy. After stirring for 5 min, a trichloromethane solution containing 50 mg triphenylphosphine (TPP) was added. After stirring for a further 10 min to allow a complete reaction, a freshly prepared aqueous solution of $NaBH_4$ (10 mg mL^{-1}) was added. After stirring for 5 h under dark conditions, a clarified red-brown solution was obtained. After centrifugation, the aqueous phase in the solution is removed and precipitated, n-hexane is added to the remaining solution for volatilization, and red rhomboid crystals can be obtained after 24 h.

Synthesis of $Ag_{13}Au_5(PTFE)_{10}(DPPP)_4$

A mixture of CF_3COOAg (8.9 mg, 0.04 mmol) and 1,3-bis(diphenylphosphino)propane (DPPP) (10.0 mg, 0.025 mmol) was stirred in the 4 mL of acetone at room temperature. Then the methylene chloride solution of Me_2SAuCl (2.9 mg, 0.01 mmol) was added and the mixed solution became slightly turbid. PTFE (6.7 μL, 0.05 mmol) was added to the above mixed solution. After stirring for 5 min, 40 μL of triethylamine was added to the above mixture with stirring. After stirring for a further 10 min for the complete reaction, a freshly prepared solution of 150 μL $NaBH_4$-ethanol solution (2 mg mL^{-1}) was added, and the colorless clear solution became a reddish-brown clear solution. After continuous stirring at room temperature and in the dark for 4 h, the resulting solid was dissolved by centrifugation with 2 mL of chloroform. After removing the solids by filtration, 2 mL of ethanol was added to the remaining solution, volatilized at room temperature and darkened for about a week to obtain red crystals. The crystals $Ag_{14}Pd$ and $Ag_{13}Au_5$ exhibit red fluorescence under 365 nm UV conditions. The excitation spectra, optimal emission spectra and fluorescence lifetime of $Ag_{14}Pd$ and $Ag_{13}Au_5$ clusters are shown in Figure S23.

1.5 Electrochemical measurements

All electrochemical tests are performed at the CH660E (Shanghai Chenhua) electrochemical workstation. The electrochemical measurements were tested in a typical gas diffusion electrode (GDE)-based flow cell with anion exchange membrane (Fumasep FAA-PK-130). Hg/HgO electrode as the reference electrode, a platinum foil electrode was used as counter electrode and catalysts modified carbon fiber paper electrodes were used as the working electrode. Both the cathode and anode compartments used 50 mL of 1 M KOH solution as the electrolyte and 200 ppm NO_3^--N reactant was added into the cathode compartment. Potentiostatic tests were performed at different applied potentials (-0.076, -0.176, -0. 276, -0. 376 -0.476 V and -0.576 V vs. RHE). All potentials are referenced to the reversible hydrogen electrode (RHE, $E_{RHE} = E_{Hg/HgO} + 0.098\ V + 0.059\ pH$).

The Faradaic efficiency (FE) is the ratio of the number of electrons transferred between the formation of products and the total current flowing through the circuit. As 16 electrons are required to form a urea molecule, and 8 electrons are required to form an NH_3 molecule, the FE of urea and NH_3 can be calculated, respectively, as follows:

$$FE_{urea}\ (\%) = (16 \times F \times C_{urea} \times V) / (60.06 \times Q) \times 100\% \qquad (1)$$

$$FE_{NH3}\ (\%) = (8 \times F \times C_{NH3} \times V) / (17 \times Q) \times 100\% \qquad (2)$$

The average yield rates of urea were calculated according to the following equation.

$$\text{Yield rate} = (C_{urea} \times V) / (t \times m_{cat}) \qquad (3)$$

where C_{urea} and C_{NH3} (g L^{-1}) are the measured urea and NH_3 concentrations, respectively; V(L) is the total volume of the electrolyte, F is the Faradaic constant (96485 C mol^{-1}), and Q (C) is the total charge passed through the working electrode; t is the time (h) for electrocatalysis and m_{cat} is the catalyst loading (g).

1.6 Products identification and quantification

The liquid products (NH_4^+, NO_2^-) were collected after electrolysis and analyzed by ion chromatography (IC, Thermo Scientific Dionex ICS-5000+). For qualitative and quantitative measurements, standard curves for NH_4^+ and NO_2^- were required.

Typically, NH_4^+-N and NO_2^--N, solutions with different concentrations in 1 M KOH solution were prepared, then 100 μL of the solution of different concentrations was removed and diluted to 5 mL, and 500 μL was injected into IC to obtain IC spectrum. By using the peak areas and ion concentrations, a linear equation for NO_2^--N and NH_4^+-N was obtained (Figure S11). Based on the measured peak areas and standard curves, the concentrations of each ion in the sample were calculated.

The gas products (CO, H_2) of electrocatalysis were analyzed by a gas chromatograph (Agilent GC7820) equipped with a thermal conductivity detector (TCD). High-purity nitrogen was used as the carrier gas, and the columns of GC are Porapak Q and MolSieve 5A. Outlet gas flow rate of electrocatalysis was controlled via a flow meter. The standard curves for CO and H_2 are shown in Figure S12.

The concentration of urea was measured by the diacetyl monoxime method. For the diacetylmonoxime method, two chromogenic solutions, A and B, were prepared. For solution A, 10 mL of phosphoric acid, 30 mL of concentrated sulfuric acid, and 10 mg of ferric chloride were added to 60 mL of deionized water; finally, deionized water was added such that the volume reached 100 mL. For solution B, 0.5 g of diacetylmonoxime and 10 mg of thiosemicarbazide were dissolved in 100 mL of deionized water. 1 mL of the post-test electrolyte was mixed with 2 mL of solution A and 1 mL of solution B and heated at 100 ℃ for 15-20 min. After cooling, the absorbance of the solution at 525 nm was measured by a UV-vis spectrophotometer.

1.7 References

1. Kresse G and Furthmüller J. Efficiency of Ab-Initio Total Energy Calculations for Metals and Semiconductors Using a Plane-Wave Basis Set. *Comput Mater Sci* 1996; **6**: 15-50.

2. Blöchl P E. Projector Augmented-Wave Method. *Phys Rev B* 1994; **50**: 17953-17979.

3. Perdew J P, Chevary J A and Vosko S H *et al*. Atoms, Molecules, Solids, and Surfaces: Applications of the Generalized Gradient Approximation for Exchange and Correlation. *Phys Rev B* 1992; **46**: 6671-6687.

4. Perdew J P, Burke K and Ernzerhof M. Generalized Gradient Approximation Made Simple. *Phys Rev Lett* 1996; **77**: 3865-3868.

5. Grimme S, Antony J and Ehrlich S *et al*. A Consistent and Accurate Ab Initio Parametrization of Density Functional Dispersion Correction (DFT-D) for the 94 Elements H-Pu. *J Chem Phys* 2010; **132**: 154104.

2. Selected spectra and data referred in the paper

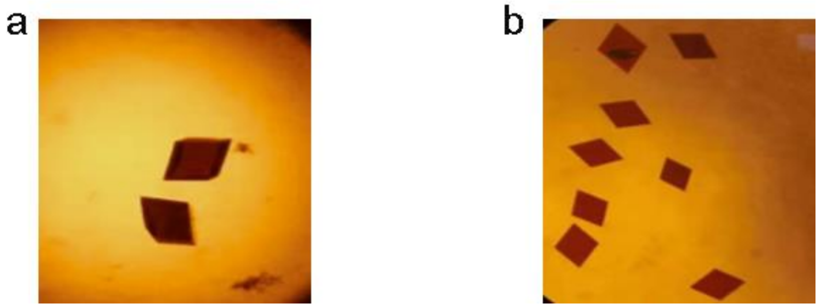

Figure S1. The photos of (a) $Ag_{14}Pd$ and (b) $Ag_{13}Au_5$ crystals under an optical microscope.

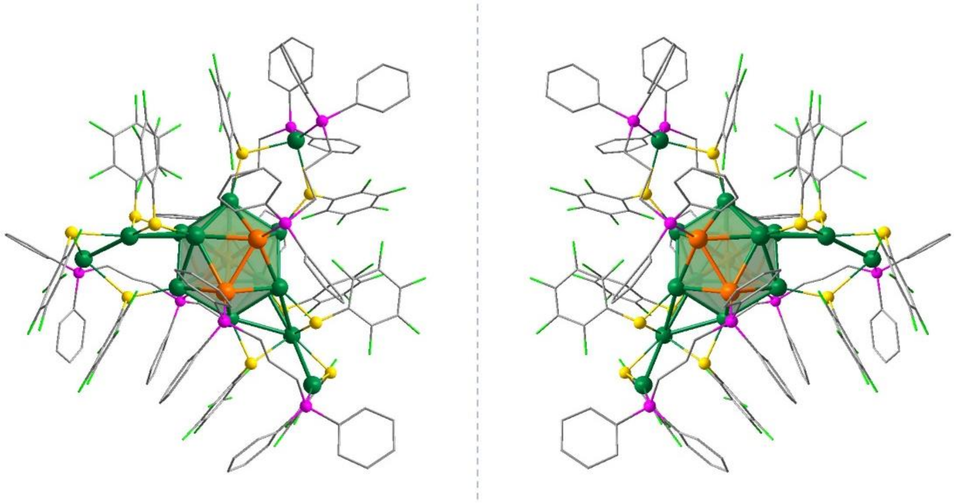

Figure S2. The enantiomer structure of $Ag_{13}Au_5$ (color code: Au, orange; Ag, green; S, yellow; P, purple; C, grey; F, bright green).

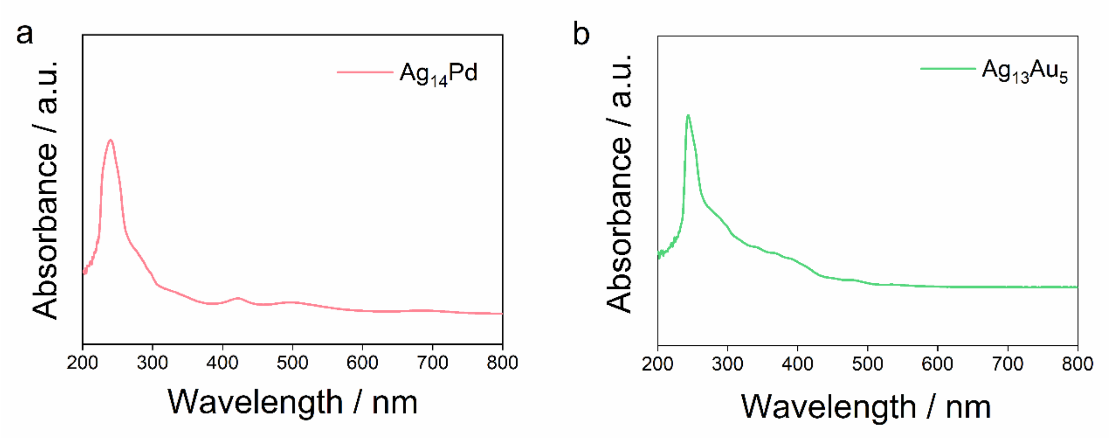

Figure S3. UV-vis spectra of (a) $Ag_{14}Pd$ in dichloromethane solution, and (b) $Ag_{13}Au_5$ in trichloromethane solution.

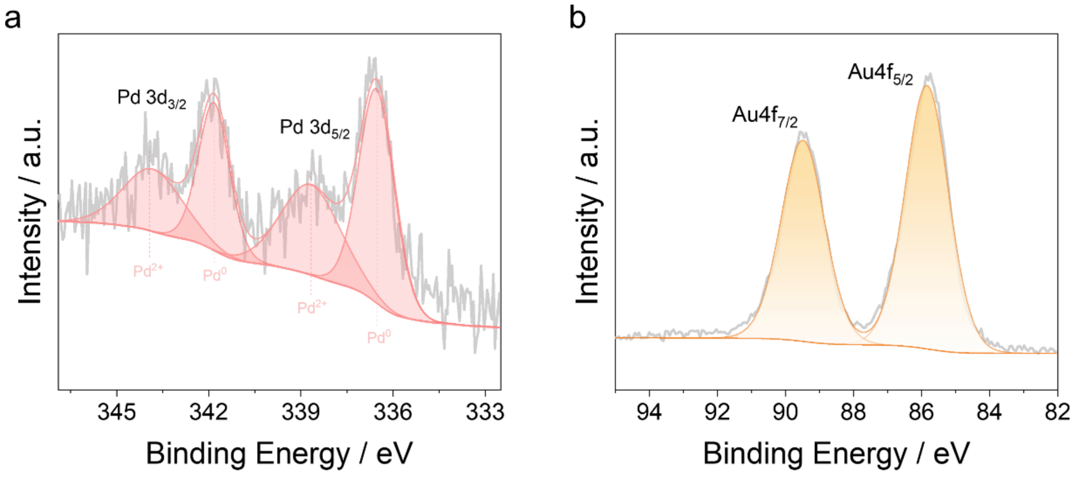

Figure S4. (a) Pd 3d XPS spectra of $Ag_{14}Pd$; (b) Au 4f XPS spectra of $Ag_{13}Au_5$.

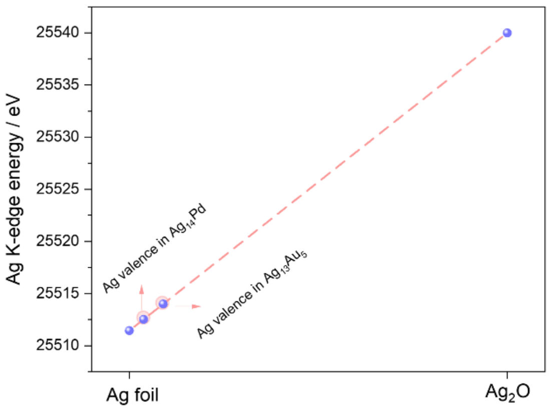

Figure S5. The calculation of Ag valence states using the edge energy determined by averaging the linear rising edge of XANES spectra.

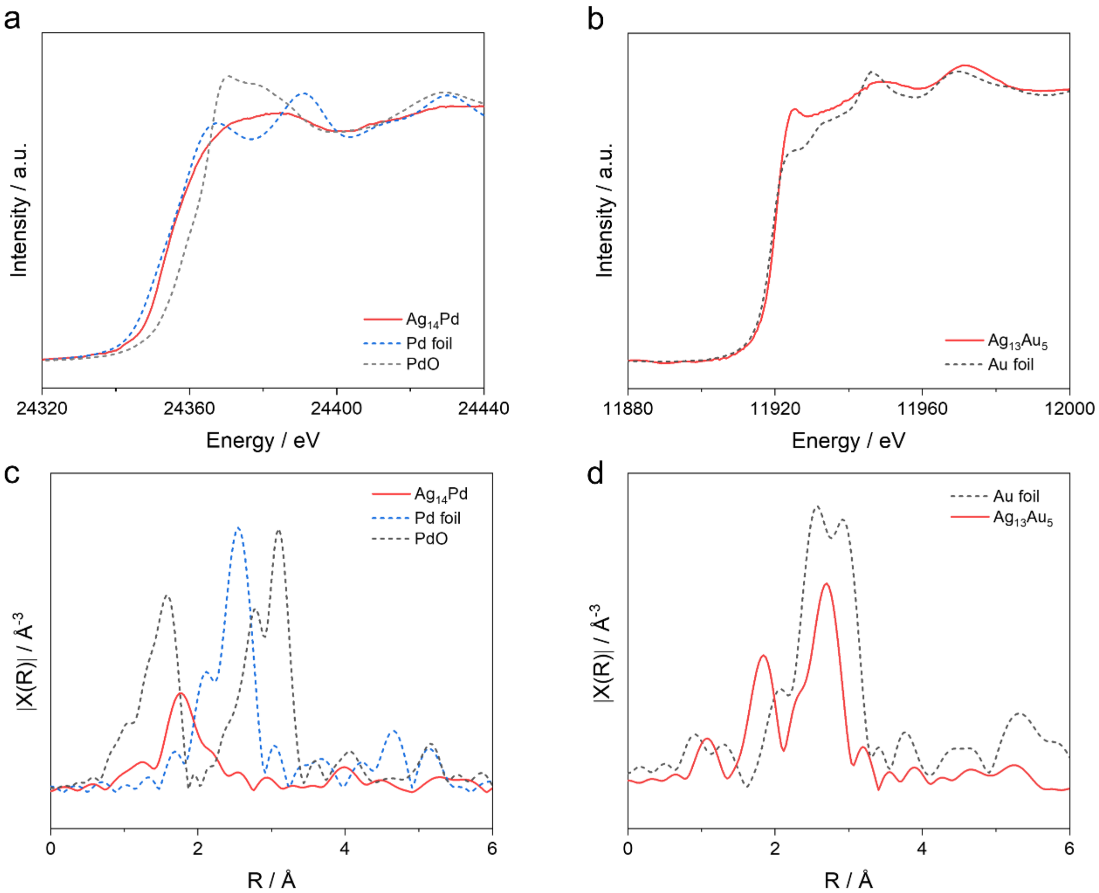

Figure S6. (a) Normalized XANES spectra of the Pd K-edge for $Ag_{14}Pd$ cluster. (b) Normalized XANES spectra of the Au L_3-edge for $Ag_{13}Au_5$ cluster. (c) k^3-weighted $\chi(k)$ function of the EXAFS spectra of the Pd K-edge for $Ag_{14}Pd$ cluster. (d) k^3-weighted χ(k) function of the EXAFS spectra of the Au L_3-edge for $Ag_{13}Au_5$ cluster.

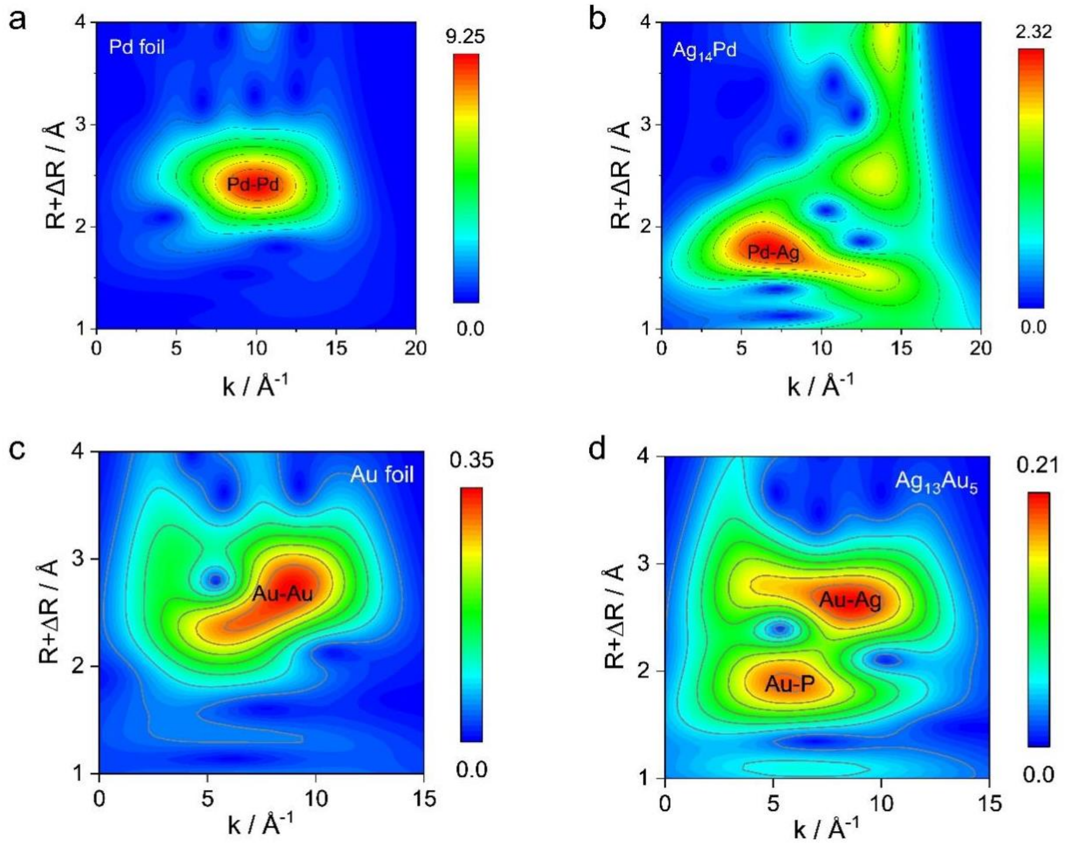

Figure S7. WT contour plots of the k^3-weighted EXAFS data. (a, b) Pd K-edge of the Pd foil and $Ag_{14}Pd$ cluster. (c, d) Au L_3-edge of the Au foil and $Ag_{13}Au_5$ cluster.

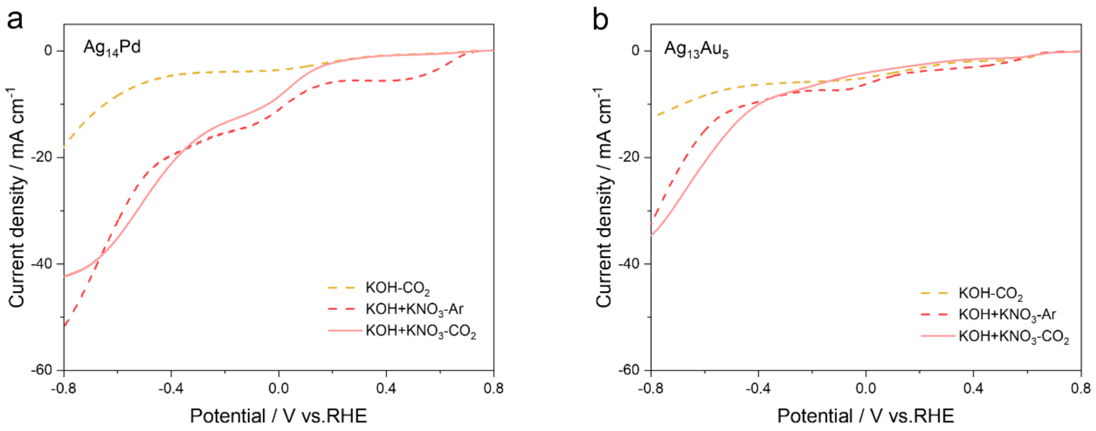

Figure S8. LSV curves of (a) $Ag_{14}Pd$ and (b) $Ag_{13}Au_5$ clusters with or without 200 ppm NO_3^--N in 1 M KOH electrolyte with Ar or CO_2 gas.

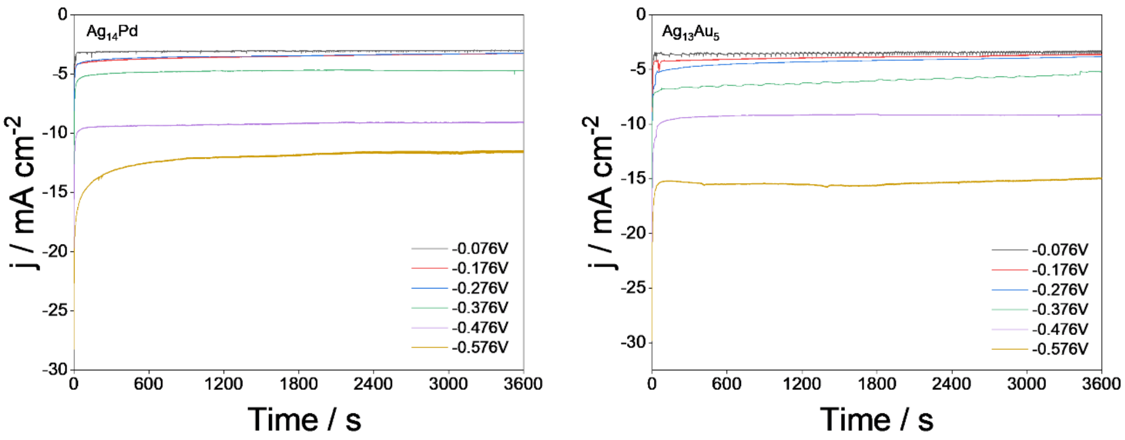

Figure S9. The i-t curves for electrocatalytic urea synthesis at various applied potentials on (a) $Ag_{14}Pd$ cluster, and (b) $Ag_{13}Au_5$ cluster.

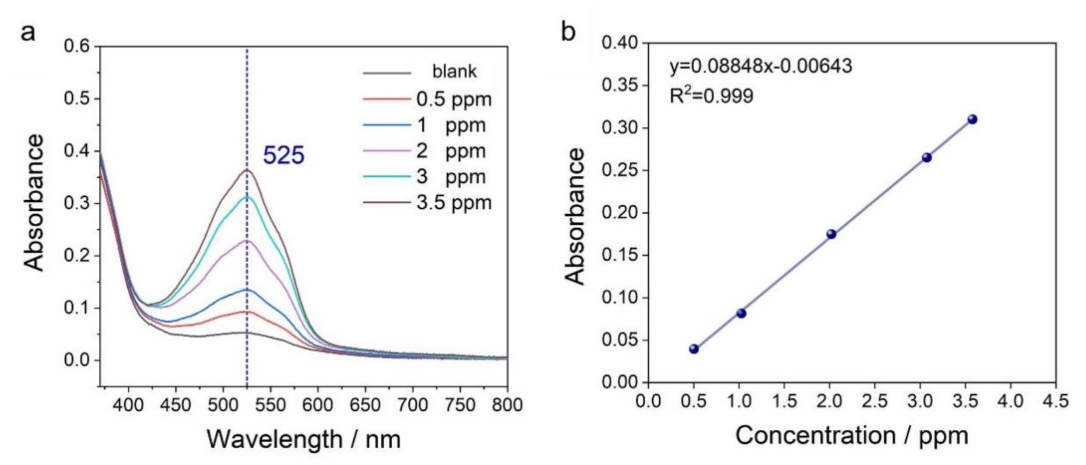

Figure S10. Calibration curve was obtained by using a known concentration of urea in 1 M KOH solution. (a) UV-vis spectra of the urea with known concentrations. (b) Calibration curve for estimating urea concentration. The absorbance at 525 nm was measured by a UV-vis spectrophotometer, and the fitted curve showed a good linear relationship between absorbance and urea concentration.

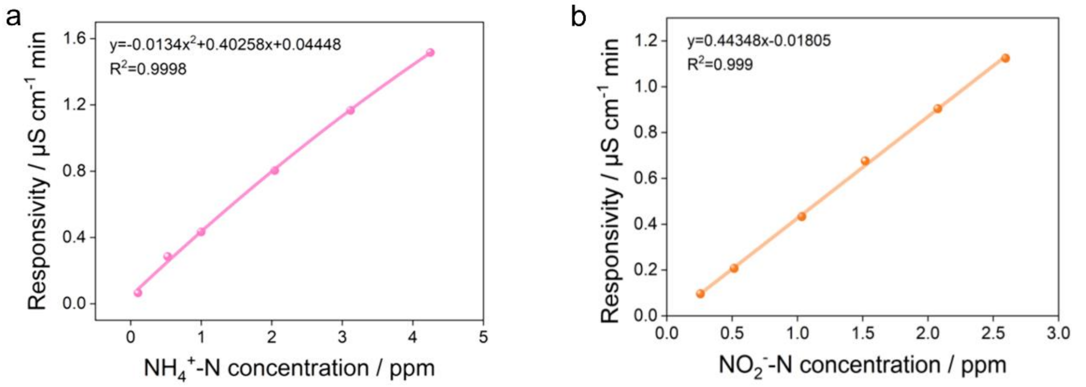

Figure S11. Calibration curves used for the quantification of (a) the NH_4^+-N concentration, and (b) the NO_2^--N concentration.

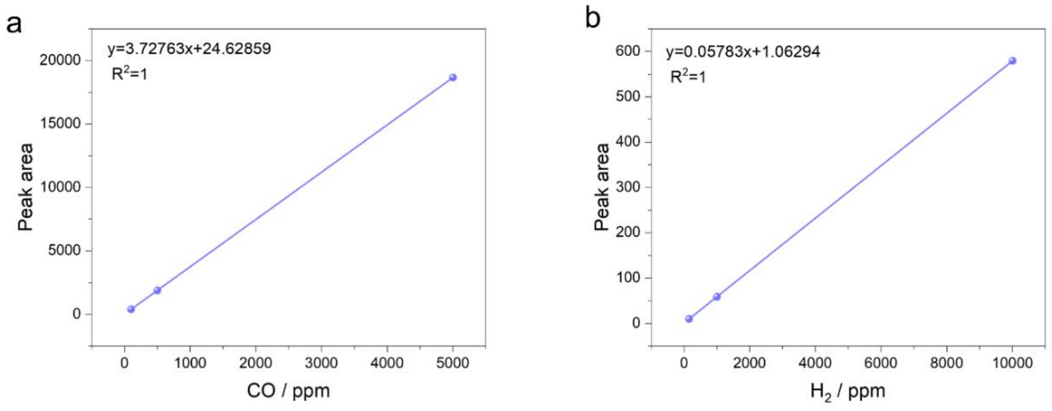

Figure S12. The calibration curve used for the quantification of (a) CO concentration, and (b) H_2 concentration.

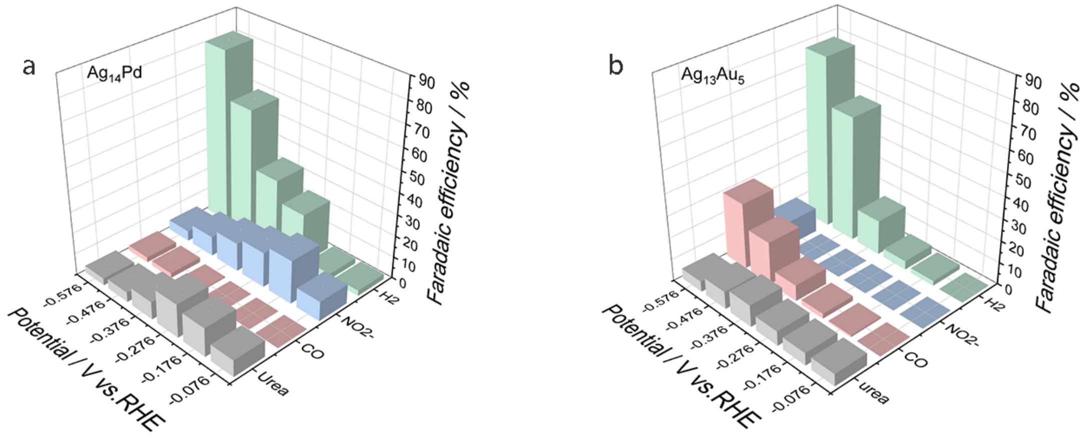

Figure S13. Faradaic efficiencies for all products at various applied potentials. (a) $Ag_{14}Pd$ cluster as the catalyst, and (b) $Ag_{13}Au_5$ cluster as the catalyst.

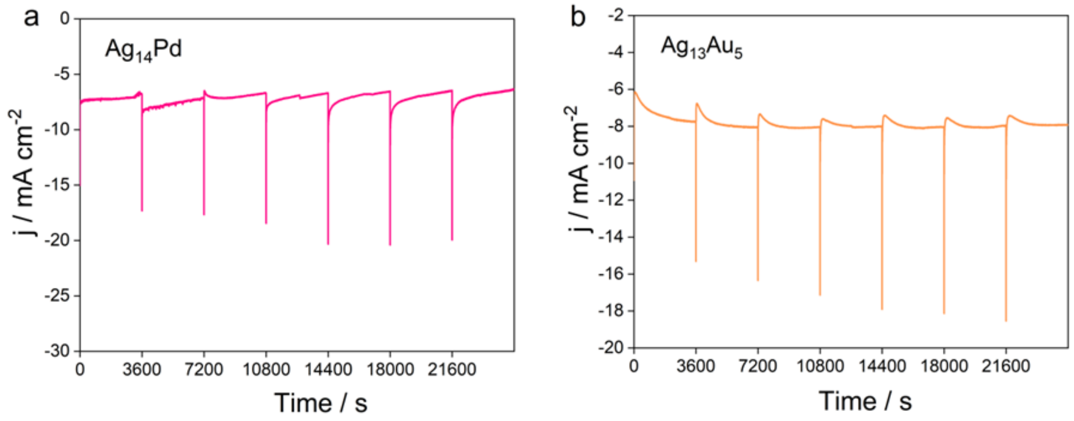

Figure S14. Stability tests of urea electrosynthesis on (a) $Ag_{14}Pd$ cluster, and (b) $Ag_{13}Au_5$ cluster at -0.276 V vs. RHE over 7 continuous cycles.

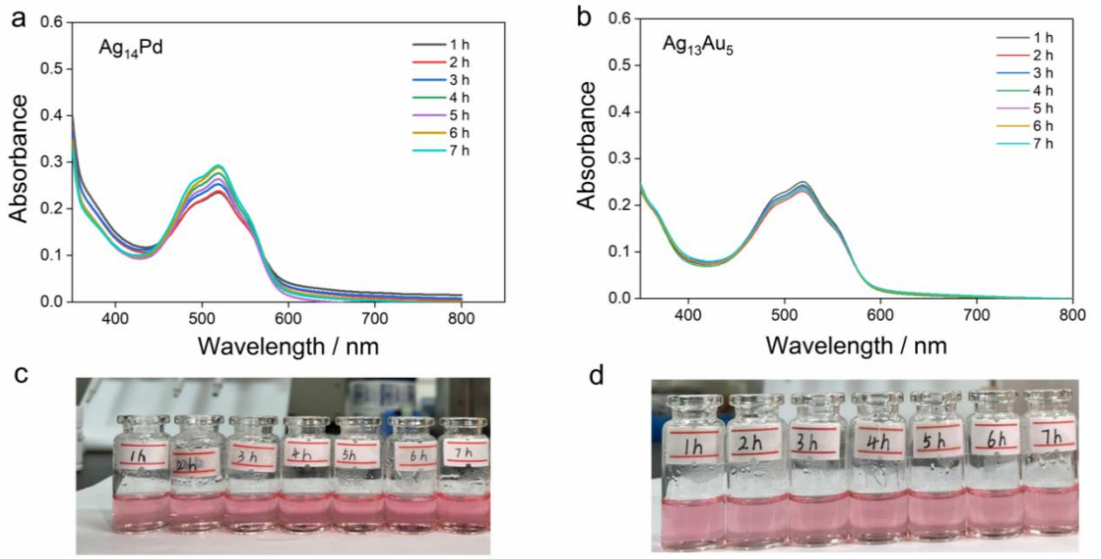

Figure S15. UV-vis spectra of (a) $Ag_{14}Pd$ cluster and (b) $Ag_{13}Au_5$ cluster for 7 cycles at -0.276 V by using diacetylmonoxime method. (c, d) Photographs of each cycle of the post-reaction electrolyte after the diacetyl monoxime method using $Ag_{14}Pd$ cluster and $Ag_{13}Au_5$ cluster as catalysts.

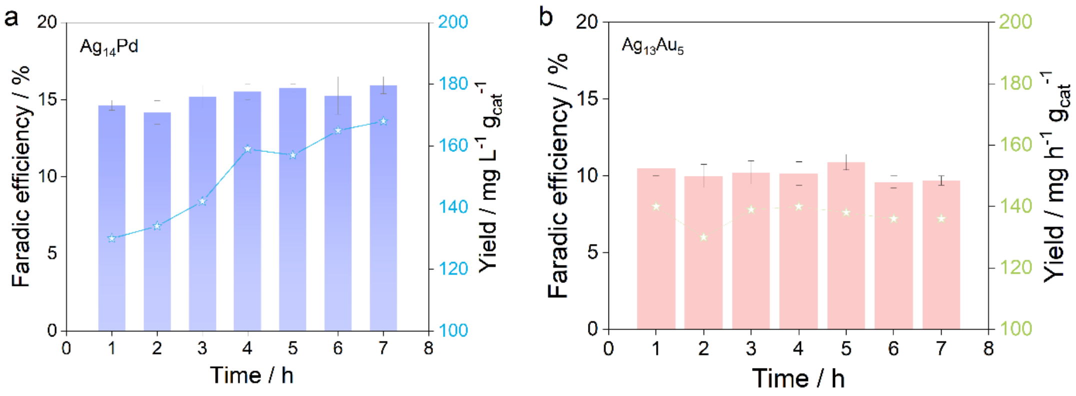

Figure S16. FEs and urea yield rate in 7 continuous cycles to reflect the stability performance of the catalysts. (a) $Ag_{14}Pd$ cluster, and (b) $Ag_{13}Au_5$ cluster.

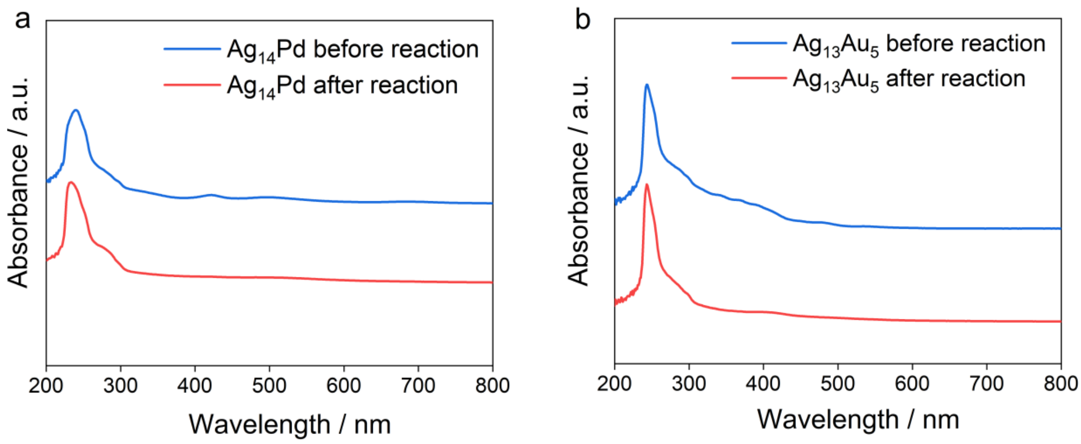

Figure S17. UV-vis of catalysts before and after reaction. (a) $Ag_{14}Pd$ cluster, and (b) $Ag_{13}Au_5$ cluster.

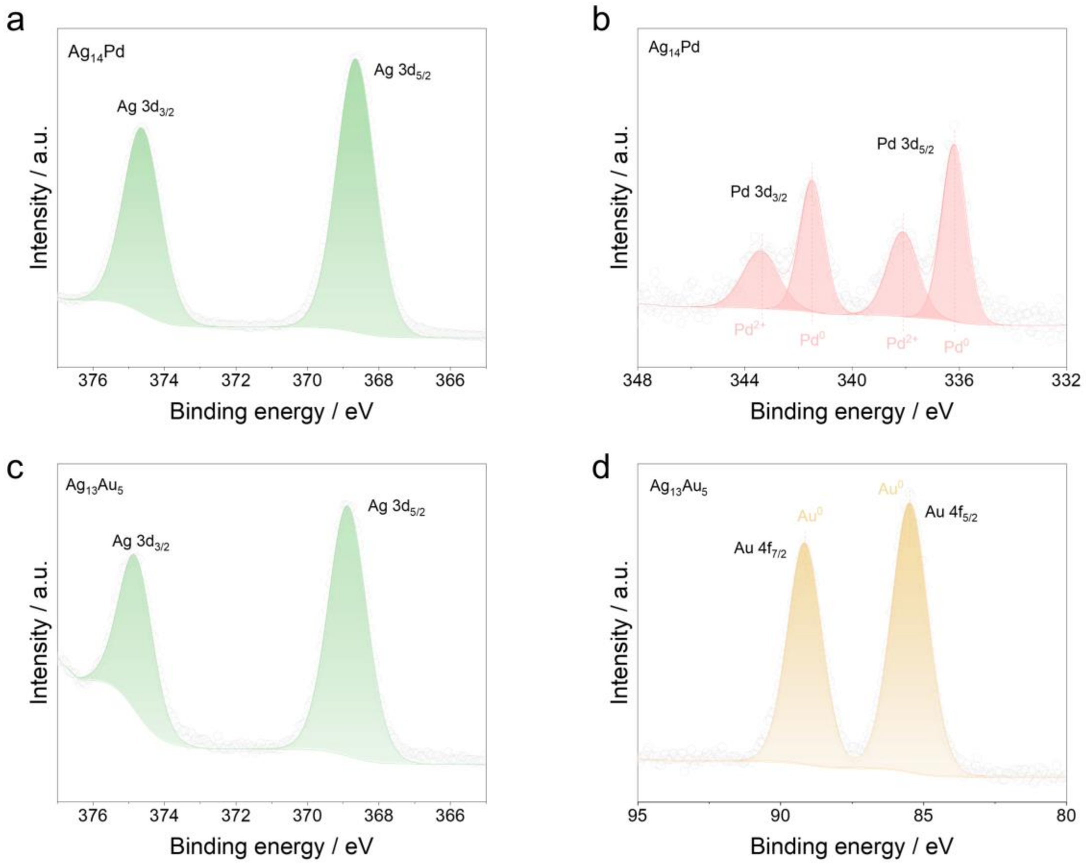

Figure S18. XPS profiles of $Ag_{14}Pd$ and $Ag_{13}Au_5$ clusters after the reaction. (a) Ag 3d XPS spectra of $Ag_{14}Pd$. (b) Pd 3d XPS spectra of $Ag_{14}Pd$. (c) Ag 3d XPS spectra of $Ag_{13}Au_5$. (d) Au 4f XPS spectra of $Ag_{13}Au_5$.

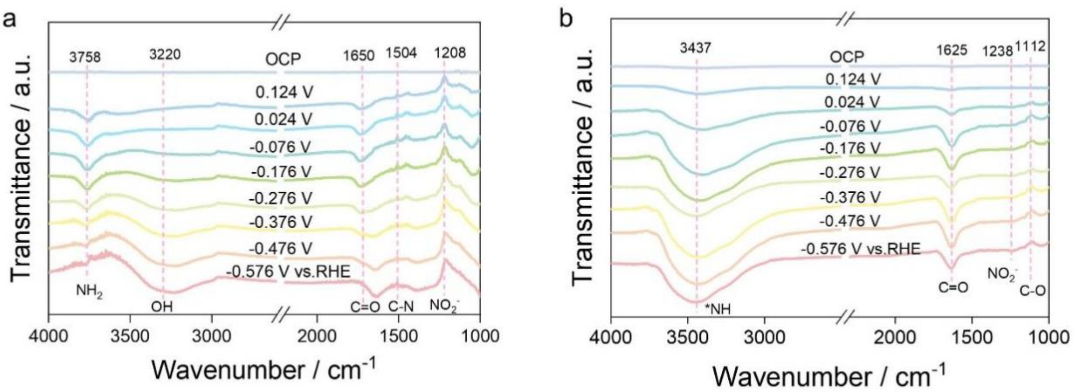

Figure S19. Potential-dependent in situ ATR-FTIR spectra of (a) $Ag_{14}Pd$, and (b) $Ag_{13}Au_5$.

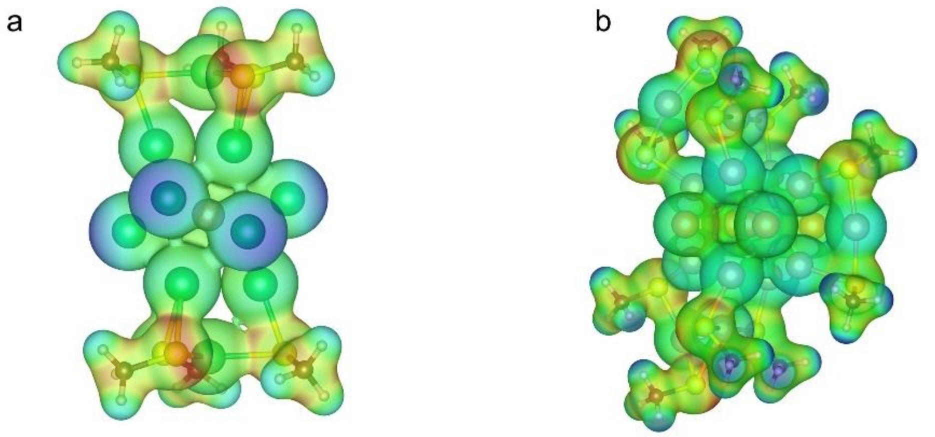

Figure S20. The charge density of (a) $Ag_{14}Pd$, and (b) $Ag_{13}Au_5$.

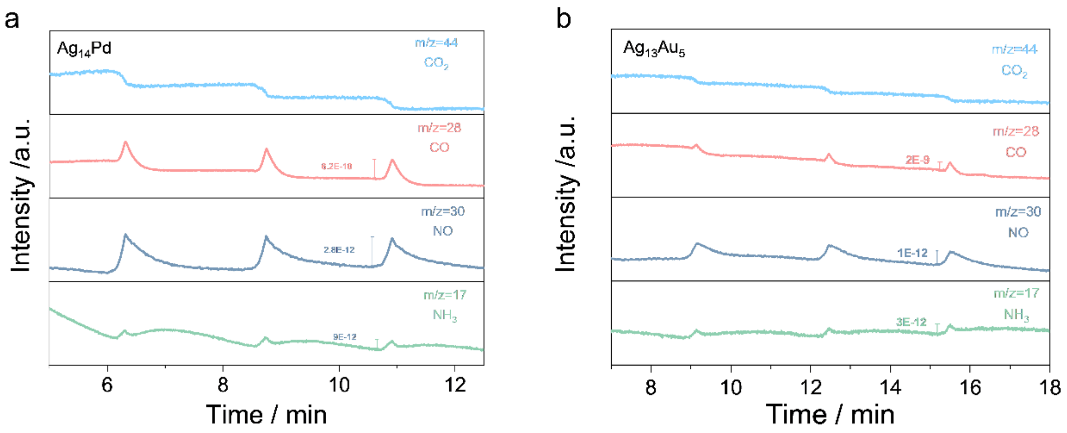

Figure S21. In situ DEMS analysis of the gaseous intermediates/products over (a) $Ag_{14}Pd$ cluster, and (b) $Ag_{13}Au_5$ cluster.

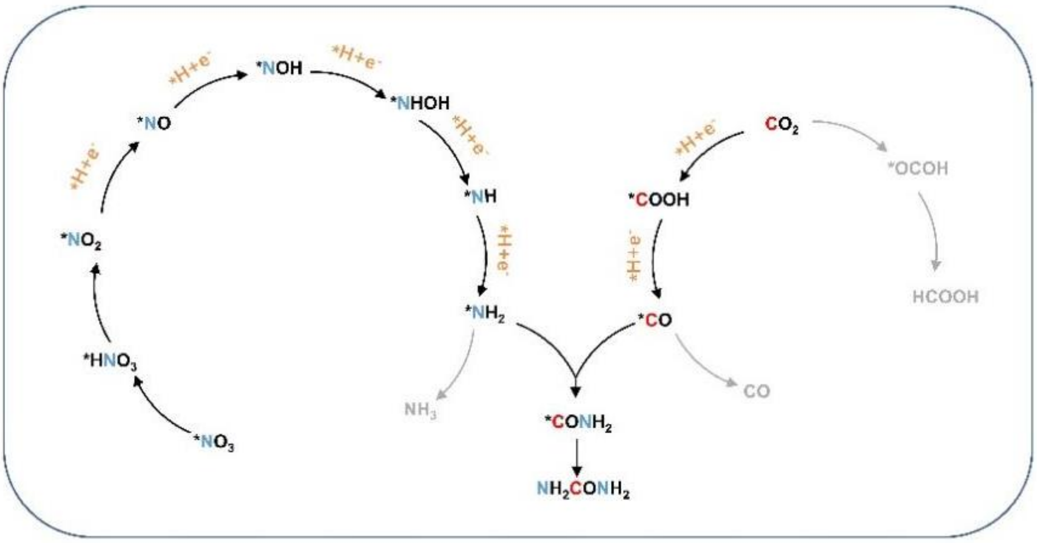

Figure S22. Urea synthesis mechanism with CO_2 and NO_3^- as feedstock in this work.

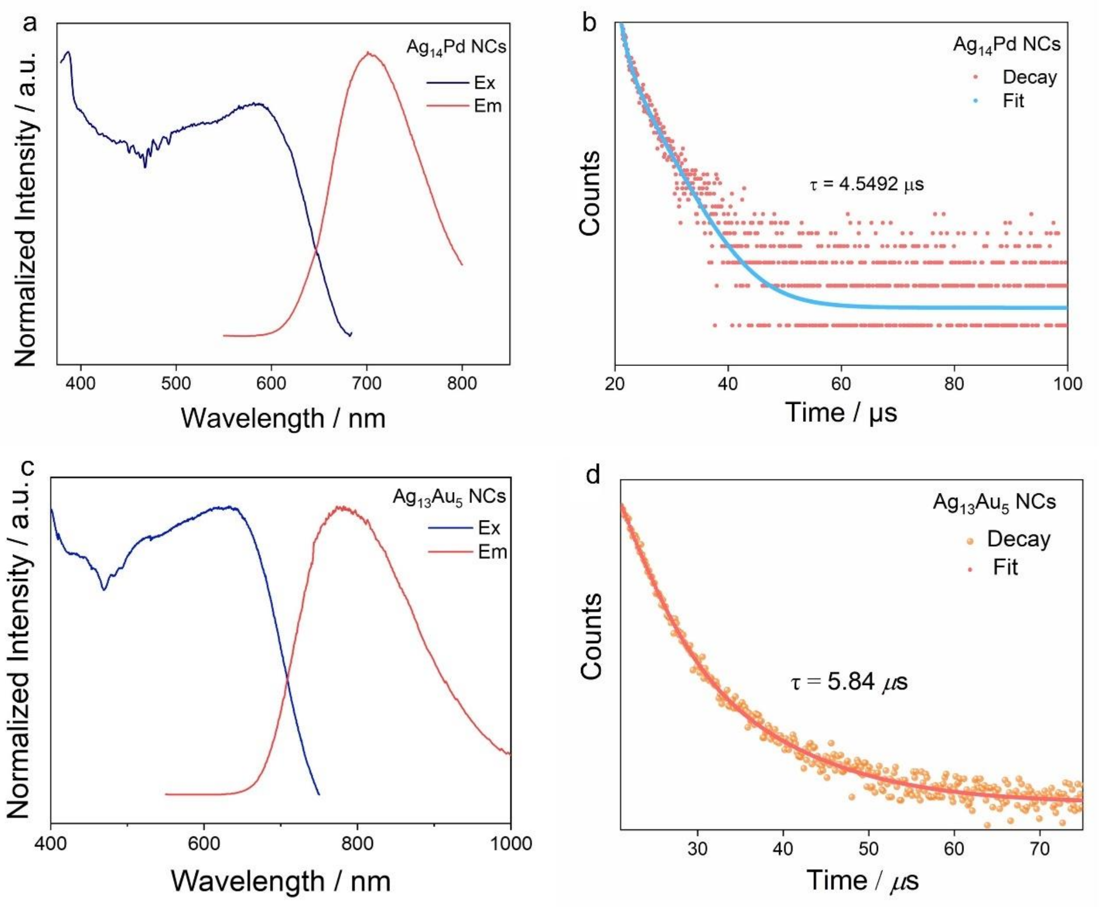

Figure S23. (a) Excitation and emission spectra of $Ag_{14}Pd$; (b) Fluorescence lifetime diagram of $Ag_{14}Pd$; (c) Excitation and emission spectra of $Ag_{13}Au_5$; (d) Fluorescence lifetime diagram of $Ag_{13}Au_5$.

Table S1. Crystal data and structure refinements for $Ag_{14}Pd$ and $Ag_{13}Au_5$.

**Compound**	$Ag_{14}Pd$	$Ag_{13}Au_5$
CCDC	2379537	2379562
Empirical formula	$C_{182}H_{122}Ag_{14}Cl_6F_{30}P_8PdS_6$	$C_{170}H_{106}Ag_{13}Au_5Cl_6F_{50}P_8S_{10}$
Formula weight	5148.19	6266.74
Temperature / K	200	200
Crystal system	monoclinic	monoclinic
Space group	*I2/a*	$P2_1/c$
a / Å	37.1610(8)	31.3885(10)
b / Å	32.0790(5)	21.8910(6)
c / Å	36.0970(7)	30.1592(8)
α / °	90	90
β / °	100.481(2)	107.210(3)
γ / °	90	90
Volume /$Å^3$	42312.8(14)	19795.3(10)
Z	8	4
ρ_{calc} g / cm^3	1.616	2.103
μ /mm^{-1}	1.612	5.287
F (000)	20048.0	11856
Crystal size / mm^3	0.2 × 0.2 × 0.1	0.08 × 0.06 × 0.05
Radiation	Mo Kα (λ = 0.71073)	Mo Kα (λ = 0.71073)
2θ range for data collection / °	4.51 to 58.818	3.78 to 57.616
Index ranges	$-50 \leq h \leq 49$<br>$-43 \leq k \leq 43$<br>$-47 \leq l \leq 44$	$-41 \leq h \leq 40$<br>$-21 \leq k \leq 29$<br>$-40 \leq l \leq 38$
Reflections collected	222951	160770
Independent reflections	51834 [R_{int} = 0.0581, R_{sigma} = 0.0572]	45868 [R_{int} = 0.1176, R_{sigma} = 0.1907]
Data/restraints/parameters	51834/12/2224	45868/664/2328
Goodness-of-fit on F^2	1.003	0.997
Final R indexes [$I >= 2\sigma(I)$]	R_1 = 0.0497<br>wR_2 = 0.1217	R_1 = 0.0741<br>wR_2 =0.1500
Final R indexes [all data]	R_1 = 0.0957<br>wR_2 = 0.1541	R_1 = 0.1764<br>wR_2 =0.1784
Largest diff. peak/hole / e $Å^{-3}$	1.12/-0.75	2.71/-1.82

$R_1 = \sum||F_o|-|F_c||/\sum|F_o|$. $wR_2 = [\sum w(F_o^2 - F_c^2)^2/\sum w(F_o^2)^2]^{1/2}$
